# Primer on Female Infertility for the Reproductive Urologist

**DOI:** 10.5152/tud.2023.23167

**Published:** 2023-11-01

**Authors:** Matthew Dullea, Joelle Mouhanna, Kyara Marquez, Akhil Muthigi, Braian Ledesma, Joshua White, Ranjith Ramasamy

**Affiliations:** 1 Department of General Surgery, University of Miami Miller, School of Medicine, Miami, Florida, USA; 2 Department of Obstetrics and Gynecology, University of Miami Miller, School of Medicine, Miami, Florida, USA; 3 Department of Urology, University of Miami Miller, School of Medicine, Miami, Florida, USA

**Keywords:** Andrology, gynecology, infertility, reproductive techniques

## Abstract

This review is intended to serve as an aid in decision-making and patient counseling for the reproductive urologist when female factor infertility is found concurrently with male factor infertility. This review pairs the pathophysiology of female infertility with its implications for the treatment of male infertility, which most commonly includes ovulatory disorders, tubal abnormalities, and uterine abnormalities. By gaining a deeper understanding of these factors, reproductive urologists can employ a tailored approach to managing male factor infertility, taking into account the female partner’s specific medical history.

**Main Points**

- Reproductive urologists need to take into consideration the female infertility factor because it may affect treatment decisions for male infertility.
- Female infertility consists of structural and hormonal abnormalities with various categories, such as ovulatory disorders, tubal abnormalities, and uterine abnormalities.
- Decisions on the treatment of infertility must take into consideration treatment success rates with the respective pathophysiology, financial burden, and desires of the patient and their partner.

## Introduction

Unlike many other conditions, infertility often affects both the patient and the patient’s partner. Per the American Society for Reproductive Medicine (ASRM), workup is recommended for infertility if pregnancy is not achieved with appropriately timed sexual intercourse after 12 months or after 6 months in women older than 35.^1^ The worldwide prevalence of infertility is up to 15%, and approximately half of these cases are attributable to male factor infertility.^1^

Given that male factor infertility (either alone or in conjunction with female factor) makes up such a large proportion of cases, the reproductive urologist is a vital member of the care team in helping a couple achieve pregnancy. Understanding the female factor of infertility can alter the andrologist’s treatment plan for the male patient. Depending on the pathophysiology and severity of infertility, treatment may improve semen parameters or sperm retrieval for in vitro fertilization (IVF). In this review, we give background on female causes of infertility and how they may affect the treatment of male infertility.

## Ovulatory Disorders

### Polycystic Ovarian Syndrome

#### Introduction:

Polycystic ovarian syndrome (PCOS) is a disease characterized by varying grades of hyperandrogenism, menstrual dysfunction, and often infertility. Polycystic ovarian syndrome affects 8%-13% of reproductive-age women, making it the most common endocrine disorder in this demographic.^2^

#### Diagnosis:

Based on a widely accepted joint consensus developed by PCOS experts in 2003 known as the Rotterdam Criteria, PCOS diagnosis must include 2 out of 3 of the following: (1) oligoovulation or anovulation; (2) clinical or biochemical evidence of hyperandrogenism; (3) polycystic-appearing ovarian morphology on ultrasound. Polycystic ovarian syndrome is a diagnosis of exclusion; thus, other disorders causing the phenotypic characteristics of PCOS should be excluded.^2^ Patient history should include a detailed menstrual history to evaluate for oligomenorrhea or amenorrhea and questions regarding features suspicious for hyperandrogenism such as hirsutism, seborrhea, or acne. On physical exam, these features may be evident as well as obesity, which can be present in up to 80% of women with this diagnosis.^3^ Biochemical markers of hyperandrogenism can be evidenced by elevated total or free testosterone. If these values are normal, dehydroepiandrosterone (DHEA) and/or androstenedione (ANSD) levels can be considered. Considering anti-Müllerian hormone (AMH) is produced by granulosa cells in ovarian follicles, women with PCOS tend to have serum AMH levels that are double or triple that of their non-PCOS counterparts.^4^ Transvaginal ultrasound can be used to assess ovarian morphology, in which polycystic ovarian morphology is consistent with 20 or more follicles per ovary or ovarian volume greater than 10 cm^3^.^2^

#### Fertility Implications and Treatment:

Polycystic ovarian syndrome is the most common cause of anovulatory infertility, representing up to 80% of cases.^5^ Other causes of infertility should be ruled out; however, if PCOS is diagnosed, nonpharmacologic measures are the first-line treatment. Lifestyle changes such as weight loss in overweight individuals (which has been shown to restore ovulatory cycles), exercise, and smoking cessation are recommended. After lifestyle modifications, ovulatory induction agents can be considered. Letrozole should be the first line for this purpose; however, clomiphene citrate and metformin can also be considered.^6^ Couples in which the female partner has PCOS should be counseled that natural pregnancy rates are lower compared to a control population and should consider IVF earlier in the course, especially if male factor infertility is present. Intrauterine insemination (IUI) can be considered in conjunction with ovulatory induction agents and may be especially helpful in cases of mild male factor infertility. However, successful IUI is postulated to require at least 5 million motile sperm to be successful, and therefore sperm retrieval procedures for IVF should be prioritized over other interventions in men with significant oligospermia or azoospermia or motility issues.^7^ It has been suggested that oocytes retrieved from polycystic ovaries have an abnormal zone pellucida structure, which can lead to decreased fertilization rates; therefore, intracytoplasmic sperm injection (ICSI) may increase fertilization rates in eggs retrieved from women with PCOS.^8^

### Diminished Ovarian Reserve and Ovarian Insufficiency

#### Introduction:

Ovarian reserve is defined as the remaining quantity of oocytes in the ovaries. In the case of diminished ovarian reserve (DOR), there is a decline in the ovarian follicular pool as well as a possible impact on oocyte quality and reproductive potential. The term may also be used as a marker for patients who may exhibit poor ovarian response during ovarian stimulation cycles.^9^ Diminished ovarian reserve is often present in women greater than the age of 35, with an acceleration in this decline when approaching ages 37-38. Besides age, there are a number of factors that may affect ovarian reserve, including but not limited to ovarian cysts (endometriomas), pelvic infections, chemotherapy and radiotherapy, smoking, obesity, and ovarian surgery.^10^

Even more detrimental for reproductive potential is primary ovarian insufficiency (POI) which is often described as amenorrhea for at least 4 months in a woman less than 40 years of age, along with 2 serum follicle-stimulating hormone (FSH) levels at least 1 month apart noted in the menopausal range. This is distinct from the binary state of menopause in that ovarian function is on a spectrum and a very small proportion of women can conceive.^11^ These patients exhibit hypoestrogenism with its associated symptoms and long-term consequences on the skeletal and cardiovascular systems.^12^ In about 90% of women with this condition, the cause is unknown; however, some described mechanisms include genetic causes (e.g., Turner syndrome), FSH orluteinizing hormone (LH) receptor mutation, enzyme deficiencies, autoimmunity, insufficient initial follicle number, spontaneous accelerated follicle loss, infections, or environmental toxin-induced follicle loss.^13^

#### Diagnosis:

Patients with DOR are typically asymptomatic; however, patients with POI will often present with some form of disordered menses prior to complete amenorrhea as well as manifestations of hypoestrogenism, including vasomotor symptoms and urogenital atrophy.^14^ When evaluating a patient with suspected POI, serum FSH should be measured twice at 4-week intervals.^15^ Beta human chorionic gonadotropin should be performed to rule out pregnancy, as should TSH and prolactin to rule out endocrine causes of menstrual abnormality. Chromosomal analysis should be performed on anyone with POI as well as fragile X premutation screening.^1^ Due to the possibility of an autoimmune nature of POI, other autoimmune disorders should be excluded. Regarding the workup of DOR, FSH levels may be considered, but elevation of FSH does not typically occur until late in the course of DOR; therefore, it is not the ideal laboratory test for this purpose. At present, AMH levels and antral follicle count (AFC) are the most sensitive markers for ovarian reserve, with lower AMH levels (<1 ng/mL) and lower AFC (<5-7) correlating with DOR.^1^

#### Fertility Implications and Treatment:

Only about 5%-10% of women with POI can conceive; thus, spontaneous pregnancy is extremely rare.^16^ Individuals with DOR also have low spontaneous pregnancy rates, so IVF is typically the treatment of choice in both cases. For few women with POI with residual ovarian reserve sufficient for ovarian stimulation, IVF with autologous oocytes may be attempted. Higher doses of exogenous gonadotropins tend to be needed, and poor responses are often observed. Thus, IVF with donor oocytes is typically recommended in the setting of POI.^17^ The prevalence of poor ovarian response (POR) among women with DOR provides a challenge even with assisted reproductive methods. Two ovarian stimulation cycles performed with maximal stimulation and poor response (low oocyte yield) are sufficient to categorize a patient with POR.^18^ Although stimulation protocols modifying the length of time, timing of medication administration, and agents used for stimulation have been attempted, no significant differences have been found in clinical pregnancy rates, and DOR remains a challenge for the reproductive endocrinologist.^19^ The male partner should be counseled that natural pregnancy rates are low in both DOR and POI; therefore, attempts to return sperm to the ejaculate, such as a reconstructive procedure for obstructive azoospermia, or to increase sperm quality in the ejaculate, such as a varicocele repair, are lower yield. In this context, sperm retrieval procedures for IVF should be prioritized.

### Endometriosis

#### Introduction:

Endometriosis is a benign and progressive disease where endometrial tissue is found outside of the uterine cavity. Endometriosis is common, with up to 10% of reproductive-age women suffering from the disease. These women experience infertility at almost twice the rate of those without the disease, and around 50% of women experiencing infertility have endometriosis.^20^ Symptoms include general pelvic pain, dysmenorrhea, dyspareunia, and infertility. Endometriosis is of great concern in reproductive medicine because it is estimated that up to 50% of women experiencing infertility have endometriosis.^21^ The disease can be difficult to diagnose because it is often asymptomatic in its early stages. Early diagnosis is important because disease progression is associated with higher rates of infertility.

#### Diagnosis:

As with any patient encounter, a clinical history and examination are essential to reaching a diagnosis. Questions that should be asked include chronic or cyclic pelvic pain, dysmenorrhea, family history, previous pelvic surgery, history of ovarian tumor, and/or dyspareunia. A physical examination with a bimanual examination is a low-risk procedure with reasonable sensitivity and specificity to detect endometriosis. The practitioner is searching for abnormalities such as pain, restricted mobility of structures, stiffness, and/or nodularity.^22^ In combination with bimanual examination, ultrasound is a useful imaging modality to aid in the diagnostic picture. Ultrasound is low-cost, low-risk, and lacks radiation exposure. Ultrasound can evaluate the endometrial cavity, lining, and possibly tubal patency.^22^ Transvaginal ultrasound (TVUS) can be beneficial for preoperative planning as well as fertility counseling.^21^

#### Fertility Implications and Treatment:

The pathophysiology of infertility due to endometriosis is complex and multifaceted. Endometriosis has been shown to cause distortion of normal anatomy, endocrine abnormalities, destruction of healthy ovarian tissue, and impaired implantation of embryos. At the simplest level, endometriosis can make natural conception too painful for the female partner. Some of the locations where endometriosis is found include the pouch of Douglas and uterosacral ligaments, which cause dyspareunia and preclude sexual intercourse.^20^

The inflammatory response caused by endometriosis can produce adhesions that distort the pelvic anatomy. These adhesions can occlude the transport of the oocyte and mechanically prevent fertilization.^23^ Endometriosis lesions are rich in Fe3^+^ and other reactive oxygen species (ROS) due in part to the inflammatory response they generate. These lesions can implant anywhere along the path the oocyte travels from the ovary and through the uterine tubes. Reactive oxygen species can damage oocytes, sperm, and embryos by inhibiting the microtubules responsible for chromosomal separation or causing DNA fragmentation.^23^ The oxidative stress can impair sperm motility, sperm acrosome reactions, and sperm–oocyte fusion.^24^

Women with endometriosis experience higher rates of implantation failure. The eutopic endometrium tissue of women with endometriosis is found to have more pro-inflammatory mediators and dysregulation of genes that would normally inhibit these mediators.^20^ The implantation difficulty can be further compounded by endocrine abnormalities. Endometriosis can alter the hypothalamic–pituitary–ovarian axis, causing distortions in the luteal phase and estrogen-progesterone balance.^24^

The most common site for endometriosis is the ovary, which presents as an endometrioma, a benign cyst without a capsule. An endometrioma can harm the ovarian reserve by mechanical damage from occupation of space, and the high concentrations of free iron may also cause oxidative stress.^20^ The difficulty of removing endometriomas can lead to further degradation of healthy ovarian tissue during surgical resection or cauterization if bleeding is experienced.^24^ Anti-Mullerian hormone, a marker for ovarian reserve, has been found to decrease following resection of an endometrioma.^21^

Because there are many mechanisms of infertility due to endometriosis, there are various treatment options and approaches. The treatment needs to be individualized based on factors such as the patient's age, stage of disease, and fertility goals. Pregnancy rates following surgery for endometriosis have been consistently reported around 50%, which compares well to those for IVF. In vitro fertilization may be the preferred method when there is male factor infertility involved as well.^25^

Patients with endometriosis experience decreased oocyte quality and implantation during IVF; however, IVF is often the best option for women with infertility due to endometriosis.^26^ It is hypothesized that the superovulation (SO) from sex steroids given for oocyte retrieval worsens the already dysregulated eutopic endometrium of women with endometriosis. A study by Chang et al 2022 found that cryopreserved embryos resulted in a statistically significant higher rate of live births compared to the transfer of fresh embryos in women with endometriosis.^27^ Deferred frozen-thawed embryo transfer may allow for a more receptive endometrium following the hyperstimulation.^27^

The pregnancy rates for women with endometriosis stage I or II are comparable to the rates with IVF. If the male factor infertility does not warrant IVF on its own, it is reasonable to offer couples with endometriosis stage I or II SO and IUI prior to IVF attempts.^28^ Evidence does not support SO/IUI in patients with severe endometriosis (stage III or IV); therefore, regardless of the male factor, IVF would be the best course of action.^28^

### Tubal Abnormalities

#### Introduction:

Tubal factors account for approximately a third of cases of infertility, and abnormalities of the fallopian tubes can be congenital or acquired.^29^ A blocked tube is defined as either an obstruction, which is transient, or a tubal occlusion, which is permanent, and both can impair the transport of sperm, ovum, or embryo, preventing fertilization or implantation. Congenital abnormalities are rare and range from agenesis to duplication of tubes, with a variety of dysmorphic anatomical differences as well.^29^

#### Diagnosis:

A history of pelvic inflammatory disease (PID), a polymicrobial infection of the upper genital tract, can make a clinician suspect a tubal cause of infertility. Salpingitis, or infection of the fallopian tubes, can result in fibrosis, causing occlusion and infertility. To evaluate a patient for tubal infertility, the clinician can utilize laparoscopy, saline infusion sonography, or hysterosalpingography (HSG). Hysterosalpongography involves the injection of a contrast medium into the uterine cavity, followed by x-ray imaging, and can help visualize the uterine cavity, fallopian tubes, and the contour of the endometrial lining. Laparoscopy is considered the gold standard for diagnosing tubal abnormalities, but HSG may be a better first-line modality given that laparoscopy is more expensive, invasive, and requires greater surgical skills. Hysterosalpingography is easier to perform, has a lower risk of complications, and has a sensitivity of 72%-85% when compared to laparoscopy.^29^

#### Fertility Implications and Treatment:

The treatment for tubal obstruction or occlusion is tubal recanalization. If a blocked tube is found on HSG, then a selective salpingography is performed on the blocked tube. Sometimes, the added pressure from selective salpingography can dislodge a blockage. If the tube remains blocked, a transcervical recanalization procedure is performed where a catheter is placed at the uterotubal junction and a guidewire is advanced through the tube. A repeat HSG is performed to assess the success of the recanalization. This procedure can provide a less invasive way of assessing and treating infertility due to tubal blockage, with an average success rate of intrauterine pregnancy following of about 30%.^1^ Even if the procedure is not successful, it serves diagnostic purposes and guides the next steps of infertility treatment.

Yuan et al developed a modified classification of tubal obstruction where, following laparoscopic surgery for tubal disease, the scoring system divides patients into categories of mild, moderate, or severe abnormalities. Follow-up over 2 years showed that infertility rates increased with higher scores. Patients placed in the mild category had an intrauterine pregnancy rate of 60.1%, while patients in the severe category had an infertility rate of 89.5%. This model can serve as a tool in counseling patients on whether they want to pursue natural conception after surgery or should consider IVF.^30^ For couples in which male factor fertility is also present, they should be counseled that the presence of unilateral or bilateral tubal obstruction significantly decreases natural pregnancy rates, and IUI may not be feasible. Therefore, attempts to improve sperm count or quality in the ejaculate, such as via a varicocele repair, have lower yield, and sperm retrieval procedures for IVF have a higher yield.

### Cervical Stenosis

#### Introduction:

Cervical stenosis, the anatomical narrowing of the cervical canal, poses a unique challenge in the realm of female infertility. The cervix plays a pivotal role in reproduction by providing a conduit for sperm transport into the uterine cavity and protecting the uterus from ascending infections.^31^ Consequently, any alteration in cervical anatomy or function can have a profound impact on fertility outcomes.

Cervical stenosis can be attributed to congenital or acquired factors.^32^ Congenital stenosis, although rare, is believed to be associated with segmental Müllerian hypoplasia.^31,32^ Acquired stenosis is predominantly iatrogenic, resulting from scarring following cervical excisional procedures like cold-knife conization and loop electrosurgical excision, with approximately 3%-9% of these procedures leading to this complication.^32^ Less commonly, causes such as infection, neoplasia, severe atrophy, and radiation changes contribute to cervical stenosis.^32^

Cervical stenosis significantly diminishes the production of cervical gland secretions, thus impeding the generation of fertile cervical mucus and compromising sperm viability, survival, and motility.^31^ The risk of infection and inflammation is increased because natural clearance mechanisms are impaired, which facilitates pathogen proliferation.^33^ Mild to moderate stenosis may still allow for natural conception, although with reduced fertility potential. Severe cervical stenosis often necessitates medical intervention or assisted reproductive techniques.

### Diagnosis:

Diagnosing cervical stenosis relies on assessing symptoms and conducting physical examinations, since there is currently no precise and universally accepted definition available. This diagnostic process can be challenging since stenosis often manifests without noticeable symptoms. If symptoms are present, they typically include dysmenorrhea, hematometra, hematosalpinx, or endometriosis.^34^ Infertility and difficulties with transcervical procedures, such as those used in assisted reproduction, are common indicators of cervical stenosis.^34^ Diagnostic methods include transvaginal ultrasound, HSG, hysteroscopy, and cervical dilation.^32,35^

## Fertility Implications and Treatment:

Treatment options include cervical dilation, cervical stenting, cervical canalization, and surgical interventions such as cervical cerclage or conization.^35^ Cervical dilation, performed using dilators of varying sizes, aims to mechanically enlarge the cervical canal, allowing for improved sperm passage.^33^ Multiple studies have reported successful pregnancies following cervical dilation, associated with improved sperm penetration and increased chances of natural conception.^32^ In cases of cervical stenosis where natural conception is challenging, assisted reproductive techniques can be considered. Intrauterine insemination can bypass the obstructed cervical canal and effectively overcome the barrier imposed by the stenotic cervix, enabling a higher concentration of motile sperm to reach the fallopian tubes, increasing the chances of successful fertilization.^33^ In cases where a patient has cervical stenosis and a male factor is also present, insemination emerges as a preferred treatment method for couples.^32,33^

### Uterine Factor Infertility

#### Introduction:

Uterine factor infertility encompasses a range of conditions that can impair a woman's ability to conceive or carry a pregnancy to term. Among the significant contributors to uterine factor infertility are endometrial polyps (EP), uterine fibroids (UF), and intrauterine adhesions (IUA). These conditions can significantly impact reproductive outcomes, necessitating a thorough understanding of their etiology, clinical manifestations, and treatment options.

#### Endometrial Polyps:

Endometrial polyps are localized overgrowths of the endometrial lining within the uterine cavity, with an incidence between 7.8% and 34.9%.^36^ A prospective study of 1000 infertile patients who underwent hysteroscopy before IVF found that 32% had EPs. The relatively high prevalence of EPs in infertile women suggests a causative relationship between the presence of EPs and infertility.^36^

#### Uterine Fibroids:

Uterine fibroids, also known as myomas or leiomyomas, are the most common type of pelvic neoplasms that affect women of reproductive age, with a cumulative incidence of approximately 70%.^37^ Their presence can disrupt the normal architecture of the uterine cavity, leading to infertility or recurrent pregnancy loss, and may be the sole cause of infertility in 2%-3% of women.^38-40^ Fibroids can cause infertility by disrupting normal endocrine function, disrupting the endometrium, and obstructing the tubal ostia, which impairs gamete transport as well as causes changes in the uterine environment that are inhospitable to sperm.^37^ These changes include alterations in the hormone composition, cytokine levels, and blood flow to the uterus and can lead to abnormalities in sperm function and motility and decreased sperm count.^39^ In addition to these physiological changes, the presence of UFs can also lead to sexual dysfunction in male partners due to physical discomfort or dyspareunia.^39^

#### Intrauterine Adhesions:

Intrauterine adhesions, also known as Asherman’s syndrome, are bands of scar tissue within the uterine cavity, often due to previous uterine surgery or infection.^41^ These adhesions can lead to significant distortion or obliteration of the uterine cavity, compromising the implantation of embryos and increasing the risk of pregnancy complications.^41^

#### Diagnosis:

Accurate diagnosis is crucial in evaluating uterine factor infertility to guide appropriate management and improve reproductive outcomes. A range of diagnostic modalities are available, each offering unique advantages and limitations, including TVUS, magnetic resonance imaging, and hysteroscopy.

Transvaginal ultrasound is often the initial imaging modality of choice due to its noninvasive nature and high sensitivity in detecting uterine abnormalities.^42,43^ It allows for the evaluation of uterine size, shape, and the presence of structural abnormalities such as UF.^43^ Transvaginal ultrasound can be enhanced with color Doppler, three-dimensional investigation, and contrast to increase diagnostic accuracy, particularly in identifying EPs.^44^ Magnetic resonance imaging offers detailed imaging and characterization of uterine pathologies, providing excellent visualization of UF, congenital uterine abnormalities (CUA), and other structural abnormalities.^39,45^ Magnetic resonance imaging is especially beneficial in surgical planning and decision-making and aids in accurate localization of pathology, particularly for intramural fibroids. Hysteroscopy, however, is considered the gold standard for diagnosing and treating intrauterine pathologies given the ability to directly visualize the uterine cavity.^41-43,45,46^ It also permits simultaneous therapeutic interventions such as polyp and fibroid removal or adhesiolysis.^39^

By employing a combination of these diagnostic modalities, health-care professionals can accurately identify and characterize uterine pathologies contributing to infertility. This enables the formulation of tailored management strategies and enhances the chances of successful reproductive outcomes for patients with uterine factor infertility.

#### Fertility Implications and Treatment

#### Endometrial Polyps:

Hysteroscopic polypectomy significantly improves fertility outcomes in women with EPs.^44^ In 1 meta-analysis of 12 studies, polypectomy increased the overall pregnancy rate from 40.4% to 56.8% and the live birth rate from 33.3% to 47.2%.^47^ Polypectomy has also been found to reduce the risk of early pregnancy loss in women with infertility and EPs and improve fertility outcomes in sub-fertile women, even in the absence of abnormal bleeding.^47^ Based on the available evidence, surgical removal is recommended to enhance the chances of natural conception or successful assisted reproductive technology.^39^

#### Uterine Fibroids:

The decision to treat fibroids in women with infertility depends on factors such as fibroid size, location, number, age, and fertility goals.^37^ For women with submucosal or intracavitary fibroids that distort the uterine cavity, myomectomy via hysteroscopy, laparoscopy, or laparotomy is recommended to enhance pregnancy rates.^40^ Pregnancy and delivery rates appear to be improved after resection of submucosal fibroids, especially when fibroids are the sole identifiable cause of infertility.^40^ The impact of intramural and sub-serosal fibroids on fertility is uncertain, and myomectomy may not be necessary for asymptomatic infertile women with non-cavity-distorting fibroids.^40,48^ Regardless of the underlying mechanism, IUI and IVF are not typically recommended as initial treatment options for infertility in women with fibroids.^38,39^ Multiple studies in the literature have demonstrated significantly lower implantation rates and clinical pregnancy rates in women with fibroids compared to women without fibroids after both IUI and IVF; therefore, treatment of fibroids is recommended prior to pursuing fertility treatment.^38,39^

#### Intrauterine Adhesions:

Intrauterine adhesions or synechiae can result in menstrual abnormalities, such as amenorrhea or oligomenorrhea, which can decrease the likelihood of natural conception. Additionally, the adhesions can impair implantation and increase the risk of pregnancy loss, particularly in cases where the adhesions involve the entire uterine cavity.^41^ Intrauterine adhesions can also affect the success of IUI by decreasing the chances of successful insemination and implantation.

Intrauterine adhesions can impair sperm transport and hinder embryo implantation by altering the shape, size, and volume of the uterine cavity. This is supported by multiple studies that show significantly lower rates of successful embryo implantation and decreased pregnancy rates in women with moderate-to-severe IUA compared to mild or no adhesions.^49^

The treatment of IUA involves hysteroscopic surgery to remove the adhesions and restore the normal uterine cavity. Mild cases of uterine synechiae may not require treatment, while more severe cases may require surgical intervention.^45^ The success of surgical treatment depends on the severity and location of the adhesions, as well as the experience of the surgeon. In a systematic review of 31 studies involving a total of 2137 patients, the overall clinical pregnancy rate after surgical treatment was found to be 49.7%, with a live birth rate of 40.5%.^45^

Given improved rates of pregnancy and delivery rates after treatment of structural uterine abnormalities, improving sperm count and quality or returning sperm to the ejaculate among couples with concurrent male factor infertility should be considered, including varicocele repair, vasectomy reversal, etc.

## Conclusion

Female factor infertility significantly contributes to the overall burden of infertility, impacting many couples worldwide who desire to conceive. It encompasses a complex array of structural and hormonal abnormalities with various categories, such as ovulatory disorders, tubal abnormalities, and uterine abnormalities, each contributing to the intricate nature of this condition. While male factor infertility contributes to a smaller percentage of cases, it remains an essential consideration in evaluating and treating infertility.

The involvement of reproductive urologists in the care team is of utmost importance, as they play a vital role in optimizing the chances of a successful pregnancy for these couples. The collaborative efforts between reproductive urologists and reproductive endocrinologists are crucial for providing comprehensive and effective management strategies for couples facing infertility challenges. The information provided in this review has been procured to offer a comprehensive overview of the common underlying conditions contributing to female factor infertility, offering insights into their implications for both natural conception and assisted reproductive technology outcomes. This individualized treatment strategy holds the potential to optimize the chances of achieving successful pregnancies for couples affected by female factor infertility. Integrating these findings into clinical practice holds great promise in significantly enhancing reproductive outcomes. It offers promising opportunities to make a profound impact on the lives of these individuals, providing them with renewed hope and the potential to experience the joys of parenthood.
